# Can policy advantages be transformed into industrial advantages? Evidences from digital economy industry development in Zhejiang Province, China

**DOI:** 10.1371/journal.pone.0298138

**Published:** 2024-03-06

**Authors:** Lin Zhou, Jianshuang Fan, Liang Ding, Zhiqian Xu, Junshen Zhang

**Affiliations:** 1 China Academy of Housing and Real Estate, Zhejiang University of Technology, Hangzhou, China; 2 School of Management, Zhejiang University of Technology, Hangzhou, China; 3 School of Design and Architecture, Zhejiang University of Technology, Hangzhou, China; 4 Innovation and Information Technology Research Branch Institute, Urban and Rural Planning and Design Research Institute Co., Ltd, Zhejiang University, Hangzhou, China; 5 ZJU Qizhen Future City Tec (Hangzhou) Co., Ltd, Hangzhou, China; Zhejiang Gongshang University, CHINA

## Abstract

The digital economy is a new impetus to promote high-quality economic development. We use the policies of Zhejiang Information Economy Development Demonstration Base (IEDD) and Zhejiang Software and Information Service Industry Base (SISI) established between 2015 and 2017 to design a quasi-natural experiment. By using a panel data from 2005 to 2020 in Zhejiang and the difference-in-differences model, we test the impacts of IEDD and SISI policies on digital economy development. We find that there are significant spatial differences for digital economy in Zhejiang. IEDD and SISI policies improve the digital economy development, that is, the policy advantages can indeed be transformed into industrial advantages. The IEDD policy can promote the digital economy industry development by enhancing the digital infrastructure and financial development; SISI policy can promote the development of the digital economy industry by promoting financial development. The results of quantile regression show that the promotion effect of IEDD and SISI policies increases with the improvement of the industrial basis of regional digital economy. The results of group regression show that the IEDD policy promotes the digital economy development in counties and county-level cities of Zhejiang, and the SISI policy plays a significant role in municipal districts.

## Introduction

With the rapid development of Information and Communication Technology (ICT) and the widespread adoption of the Internet, human society has gradually entered the digital economy era. The digital economy generally refers to various new economic forms based on digital technology [1, 2]. Its concept is not static but continuously expands with the evolution of digital technology. With the rapid development of a large number of disruptive digital technologies such as the mobile Internet, Internet of Things, big data, cloud computing, and artificial intelligence, the digital economic industry characterized by leading-edge technological features is also constantly changing [3, 4]. The digital economy reduces transaction barriers and market frictions, and the digital economy industry development transforms the methods of production, consumption, and distribution within the national economy, thereby creating value for enterprises and facilitating a more efficient model of economic operation for society [5, 6].

Therefore, the digital economy industry has become a key driving force for global economic growth. The European Union (EU), the Asia-Pacific Economic Cooperation (APEC), the World Trade Organization (WTO), the World Bank, the Organization for Economic Cooperation and Development (OECD), and countries such as the United States (U.S.), Germany, and China, perceive the development of digital economy industry as crucial to ensuring prosperity and maintaining a competitive edge. The U.S. is the first country to formulate plans regarding the digital economy. Since 1998, the U.S. Department of Commerce has published a series of reports on the “Emerging Digital Economy.” In recent years, it has unveiled the U.S. Digital Economy Agenda and the U.S. Global Digital Economy Grand Strategy. In 2010, Germany published its “The ICT Strategy: Digital Germany 2015.” In 2016, China issued the “National Informatization Development Strategy Outline.” In 2017, General Secretary Xi Jinping highlighted the importance of the digital economy development and issued significant directives during the World Economic Forum Annual Meeting and the “Belt and Road” International Cooperation Summit Forum. Zhejiang Province is a pioneering region in China’s digital economy, strongly prioritizing the advancement of the digital economy industry. In 2014, Zhejiang Province prioritized the information economy at the forefront of seven key industries, each with the potential to reach a trillion yuan in development, and pioneered with the issuance of “Guiding Opinions on Accelerating the Development of the Information Economy”, outlining plans for distinctive and leading e-commerce, the Internet of Things, cloud computing, big data, financial technology, smart logistics, and a digital content industry hub. In the same year, Zhejiang released the nation’s inaugural “Information Economy Development Plan (2014–2020)”, which further clarified the overarching principles and development objectives for Zhejiang’s information economy industry. In addition, in 2015 and 2017, Zhejiang province issued the establishment of Zhejiang Information Economy Development Demonstration Base (IEDD) and the Zhejiang Software and Information Service Industry Base (SISI), aiming to promote the rapid development of information economy industry in Zhejiang and realize the integrated development of digital economy industry and entity economy.

However, the capacity of a country or region to harness the pulse of the digital economy and translate potential policy advantages into tangible industrial strength represents a complex and formidable challenge. An adequate and proactive policy framework is essential to foster the successful advancement of the digital economy sector, ranging from infrastructure construction, establishment of a regulatory framework, and tax incentives, to education and skill development, enhancing market accessibility and ensuring equitable competition, as well as fostering international cooperation and trade promotion. The development and execution of these policies collectively cultivate an ecosystem favorable to innovation in digital technology and industrial advancement. However, to actualize this leap requires not only strategic foresight and diligent implementation from policymakers but also a proactive stance and sustained innovation within the industry. This study focuses on counties within Zhejiang Province, China, as its research subjects, and thoroughly investigates how digital economic policies (IEDD and SISI pilot polices) are ultimately mapped into competitive advantages in the industrial field through the implementation of a series of strategies, as well as the driving factors in the process of realizing this transformation.

The central government and the provincial government of Zhejiang have made substantial investments in the development of the digital economy industry. According to the “Zhejiang Internet Development Report in 2021”, the added value of core digital economy industries in Zhejiang amounted to 834.8 billion RMB in 2021, accounting for 11.4% of the national gross domestic product (GDP). In 2021, Zhejiang’s new service index for digital life grew by an annual rate of 23.7%, the highest among all Chinese provinces, with Hangzhou topping the list among 335 cities in China. Nevertheless, the influence and modalities through which digital economy policies affect the development of the digital economy industry remain ambiguous. To quantify the influence of digital economic policies on industrial arrangements would furnish novel insights and theoretical stimuli for the expansion of industrial economics while offering a referential basis for governmental bodies to both establish and appraise industrial strategies.

Utilizing data sourced from the county-level administrative divisions in Zhejiang from 2005 to 2020, we conduct a detailed examination of the spatial-temporal dynamics characterizing region’s digital economy industry progress. Then we use the IEDD and SISI policies to design a quasi-natural experiment to study the impact of digital economic policies on digital economy industry development. Our findings illuminate pronounced spatial heterogeneity within the digital economic landscape across Zhejiang’s counties. The county-level administrative regions in Hangzhou are the core circle of digital economy development, boasting a notably dense industrial high-high agglomeration, whilst peripheral districts have yet to manifest significant high-high clustering tendencies. We further find that both the IEDD and SISI policies improve the development level of digital economy industry, promoting the development level of digital economy industry to increase by an average of 25.9% and 41.4%, respectively. Variegated analysis outcomes underscore the efficacy of the IEDD and SISI policies in fostering the ascent of digital industrial within Zhejiang. This means that policy advantages can be transformed into industrial advantages. The results of the mechanism analysis indicates that IEDD policy promote the digital economy industry development by enhancing digital infrastructure and financial development; SISI policy can promote the development of the digital economy industry by promoting financial development. The results of the heterogeneity analysis show that the IEDD policy promotes the digital economy industry development in county-level cities, and the SISI policy plays a significant role in promoting the digital economy industry development in municipal districts. The promotion effect of IEDD and SISI policies on the digital economy industry development increases with the with the improvement of the industrial basis of regional digital economy.

This study enriches the literature in three ways. First, we select the Zhejiang county-level districts as our subjects for study, assessing the development level of the digital economic industry from this regional perspective based on a large number of micro-enterprise samples, and discussing its characteristics and spatiotemporal evolution with meticulous granularity. Second, we evaluate the impact of digital economy policies on the digital economy industry and explore the channels through which these policies exert their influence. Third, we deliberate on the differing roles played by two types of demonstration-based policies under varying levels of digital economic industry development and across diverse regions.

The rest of the paper is structured as follows. First, we introduce the policy background and literature. Second, we specify the model and describe the data. Third, we demonstrate the spatial-temporal characteristics of the digital economy development in Zhejiang. Fourth, we present and discuss the empirical results. Finally, there are concludes.

## Policy background and literature review

### Policy background

Since 2015, to promote digital Zhejiang and accelerate the development of digital economy industry, Zhejiang Provincial Commission of Economy and Informatization and Zhejiang Provincial Finance Bureau have carried out the establishment of IEDDs. The Zhejiang Provincial government gathers resources, optimizes the development environment, and promotes the clustering and innovation development of various information economy enterprises, institutions, and talents to demonstration bases to form a batch of demonstration bases with significant influence and demonstration and leading roles and promote the rapid development of digital economy and economic and social transformation upgrading in Zhejiang. The funding period for the demonstration base is three years. There are 12 demonstration bases in the first batch, and the funding period is from 2015 to 2017, with a subsidy of 4 million yuan per year. There are 12 demonstration bases in the second batch, and the funding period is from 2017 to 2019, with a subsidy of 3.1 million yuan per year. Table 1 presents the details of the IEDDs.

**Table 1:** IEDD policy.

Years	Regions selected as IEDD	Funding
First batch (2015–2017)	West Lake District of Hangzhou City, Binjiang District of Hangzhou City, Yuhang District of Hangzhou City, Yinzhou District of Ningbo City, Cixi City, Yueqing City, Deqing County of Huzhou City, South Lake District of Jiaxing City, Tongxiang City, Shangyu District of Shaoxing City, Gaoxin (Wucheng) District of Jinhua City, Wenling City	Support the selected bases for three years (2015–2017), with a subsidy of 4 million RMB per year.
Second batch (2017–2019)	Xiaoshan District of Hangzhou City, Zhenhai District of Ningbo City, Yuyao City, Longwan District of Wenzhou City, Haining City, Wuxing District of Huzhou City, Yiwu City, Yongkang City, Jiaojiang District of Taizhou City, Zhoushan New Town (Dinghai District)	Support the selected bases for three years (2017–2019), with a subsidy of 3.1 million RMB per year.

At the same time, Zhejiang promulgated the “Software and Information Service Industry Base (Park, Town) Construction Plan in Zhejiang Province”, and 17 county-level administrative regions were identified as the SISIs of Zhejiang Province in 2016 and 2017, including demonstration bases, characteristic bases and innovation bases. The one-time support fund for the demonstration bases is 10 million yuan; that for characteristic bases is 6 million yuan; and that for innovation bases is 3 million yuan. Table 2 shows the details of SISIs. By sorting out the policies, we do not find that the establishment of IEDDs and SISIs is subordinate to other digital economy policies, which is conducive to excluding the interference of other policies. In addition, the IEDD and SISI policies have the characteristics of promoting in batches, which provides quasi-natural experiment conditions for evaluating policy effects.

**Table 2:** SISI policy.

Years	Region selected as demonstration bases (a one-time support fund of 10 million yuan)	Region selected as characteristic bases (a one-time support fund of 6 million yuan)	Region selected as innovation bases (a one-time support fund of 3 million yuan)
First batch (2016)	Binjiang District of Hangzhou City, Yuhang District of Hangzhou City, Xihu District of Hangzhou City, Jinhua Development Zone (Wucheng District), Yinzhou District of Ningbo City, Gaoxin (Yinzhou) District of Ningbo City	Jindong District of Jinhua City, South Lake District of Jiaxing City, Xiuzhou District of Jiaxing City, Lishui City (Liandu), Lucheng District of Wenzhou City, Haishu District of Ningbo City	Gaoxin (Yuecheng) District of Shaoxing City, Wuxing District of Huzhou City, Kaihua County of Quzhou City, Zhoushan Marine Science City (Dinghai District), Xinchang County of Shaoxing City
Second batch (2017)	Xiacheng District of Hangzhou City, Jianggan District of Hangzhou City, Xiaoshan District of Hangzhou City	Gongshu District of Hangzhou City, Yueqing City of Wenzhou City, Jiashan County of Jiaxing City, Wucheng District of Jinhua City	Longwan District of Wenzhou City, Deqing County of Huzhou City, Anji County of Huzhou City, Jiaxing Economic and Technological Development Zone (Xiuzhou District), Haiyan County, Tongxiang City, Jinyi Urban New (Jindong) District of Jinhua City, Yongkang City, Jiaojiang District of Taizhou City, Economic Development Zone (Jiaojiang District) of Taizhou City

### Literature review and research hypotheses

#### Calculation of digital economy

The quantitative measurement of the digital economy has become an important focus of academic research. Scholars initially use the value-added method to measure the digital economy [7–9]. For example, the U.S. BEA measures the scale of the information economy, its added value, and the contribution of the digital economy in the national from a value-added framework [10]. Although the value-added method can gauge the scale of the digital economy, wing to the varied interpretations of its essence, the applied methodologies diverge, leading to variations in the computational outcomes. Therefore, numerous scholars and institutions establish a comprehensive index system and adopt mainstream comprehensive evaluation models, such as the TOPSIS method, the entropy value method, and principal component analysis method to measure the scale of the digital economy [11–13]. For example, Zhang et al. (2021) [4] construct an evaluation index system for digital economic industry development from three facets: infrastructure, industrial, and integration, including 12 indicators, to compute the digital economy development index across 30 provinces in China. Investigations into quantifying China’s digital economy predominantly occur at the national and provincial tiers, with fewer studies aimed at the city level and scant attention to the county level. This provides an opportunity for marginal contribution to this paper.

#### The impact of policies on the digital economy industry

In situations where the digital economy is relatively underdeveloped, relying exclusively on the market for regulation might be insufficient. Government policy intervention can provide guidance, enhance capabilities, and offer protection [14]. The digital economy policy aims to promote, standardize, and govern the development of digital economy industry. This primarily involves the content of basic theoretical research and technological breakthroughs, infrastructure construction and information resource development, result transformation and application integration, policy regulations, and cyber security. Their purpose is to enhance the level of regional digital economy industry development by building a sound public service mechanism and an innovative entrepreneurship environment. Although the evaluation of the outcomes stemming from the implementation of digital economic policies remains insufficiently explored. In developed nations, research has primarily concentrated on the outcomes associated with the implementation of broadband policies. In Chile, a study indicates that the broadband price subsidy policy only promotes the Internet application of some households, thus, comprehensive policy measures including fostering computers and Internet application development, enhancing digital literacy training need to be simultaneously considered [15]. In the U.S., state-level funding programs have a significantly positive impact on general (and fiber) broadband availability [16]. Belloc et al. (2009) [17] conduct an empirical study on OECD countries and find that both the institutional environment and demand-side intervention policies facilitate the application of broadband networks. The proliferation and utilization of high-speed internet connectivity serve as the cornerstone for the advancement of the digital economy industry. Through well-crafted digital economy policy, the government can steer the development trajectory of the digital economy industry, clarify the areas of priority development, pivotal technologies, and applications to be promoted within the sector and assist in the formulation of industrial plans and corporate strategies. Furthermore, for IEDD and SISI policies, the government offers incentives, including financial support and expedited pathways for talent acquisition, which can catalyze private investment in technological innovation and the digital economy industry. Thus, we propose the following hypothesis:

Hypothesis 1: The implementation of IEDD and SISI policies promotes the development of digital economy industry in Zhejiang.

Government investment in pilot projects for the digital economy typically encompasses R&D funding and modernization of the digital infrastructure. The development of digital infrastructure has propelled the rapid growth of information-based industries, laying the foundation for enhancing the innovative capacity of the digital economy industry. The construction of digital infrastructure effectively breaks the constraints of time and space in information exchange, accelerating the spread and communication of information and knowledge among various innovative entities. This not only broadens channels and means for labor to acquire education, creating conditions for skill improvement and overall competence enhancement, but also reduces the search and transmission costs of external knowledge in enterprise research and development. It provides opportunities for promoting collaborative research and technological exchange among businesses, and facilitates the formation of a knowledge-intensive innovation network. Bukht and Heeks (2018) [18], Gruber (2019) [19], and Zhou (2022) [20] both emphasize the importance of digital infrastructure in driving digital economic growth, with Gruber (2019) [19] specifically highlighting the need for policies that strengthen the intangible asset base and R&D activities. Not only the digital economy policies in Zhejiang Province, but policymakers worldwide are actively seeking to harness the potential of digital connectivity in order to drive economic growth and enhance living standards [21]. Thus, we propose the following hypothesis:

Hypothesis 2: The IEDD and SISI policies can promote the digital economy industry development by enhancing the digital infrastructure in Zhejiang.

The elongated duration of corporate digitization transformation, coupled with the elevated demand for digital infrastructure, engenders heightened costs and risks, thereby subjecting enterprises to formidable financial constraints during their digital transformation journey [22]. The implementation of digital economic policies has expanded the financing channels and models for businesses, accelerating the circulation speed of capital and reducing the cost of financing for enterprises, thereby enhancing the development level of the financial market. Luo (2022) [22] and Yao and Yang (2022) [23] both find that finance development can alleviate financing constraints and drive innovation in the digital transformation of enterprises. This is further supported by Fan et al. (2022) [24], who demonstrated that digital finance can improve the financial environment for green technological innovation. These findings collectively underscore the positive impact of financial development, particularly in the digital sphere, on digital economic growth and the digital transformation of businesses. The enhancement of the level of financial development provides financial security for the development of the digital economy industry. The improvement of the level of financial development promotes the optimal allocation of financial resources in the digital innovation field, thereby driving the development of the regional digital economy [25, 26]. Thus, we propose the following hypothesis:

Hypothesis 3: The IEDD and SISI policies can promote the digital economy industry development by improving the level of financial development in Zhejiang.

## Methodology and data

### Empirical model

We use difference-in-differences (DID) model to estimate the causal effects of the IEDD and SISI policies on the digital economy industry development in Zhejiang. Because the IEDD and SISI implementation at different times in each county-level administrative region, some county-level administrative region may belong to the control group at time *t*, and belong to the treatment group at time *t*+1. This highlights the need for a multi-period DID model, which is set as follows:

$$Y_{ijt}=\alpha_{0}+\beta_{1}IEDD_{ijt}+\beta_{2}SISI_{ijt}+\lambda X_{ijt}+\gamma_{j}+\mu_{t}+\varepsilon_{it}$$ (1)

where *i*, *j* and *t* denote prefecture-level city, county-level administrative region and year, respectively; *Y* denotes the digital economy industry index; *IEDD* and *SISI* are dummy variables that equal 1 in the years after county-level administrative region *j* implement IEDD and SISI policies, respectively, and 0 if otherwise; *X* represents a series of control variables, including ln*Pgdp* (regional economic development level), ln*Pop* (regional population size); *Secondary* (secondary industrial structure), *Tertiary* (tertiary industry structure), *Fixed* (capital investment), and *Patent* (regional innovation level); *γ*_*j*_ is the fixed effect of county-level administrative region; *μ*_*t*_ is the year fixed effect; *ε*_*it*_ is the random error term.

### Variables and data

1. Dependent variable. We draw on the research of Zhang et al. (2021) [4] to construct an evaluation index system to measure the development of the digital economy industry in counties in Zhejiang from the two dimensions of digital economy enterprise scale and digital economy enterprise investment scale. It includes four secondary indicators, including the number of digital economy enterprises, total registered capital, total outward investment, and total accepted investment. Then, we use the principal component analysis method to calculate the digital economy industry development index to measure the development level of the digital economy industry. We collect data on all industrial and commercial enterprises in Zhejiang from the Tianyancha website (https://www.tianyancha.com/). As of April 2021, Zhejiang was home to 3.772 million registered industrial and commercial enterprises. Subsequently, we define digital economy enterprises based on the categorization of digital economy industries in Zhejiang, and finally screen out 289,000 digital economy enterprises. The total registered capital, total outward investment, and total accepted investment for each digital economy enterprises are collected. Finally, we match the enterprise data to county-level administrative regions in Zhejiang based on geospatial data.
2. Independent variable. The independent variables are *IEDD* and *SISI*. We obtain the information of IEDD and SISI policies from the official website of the Zhejiang Provincial Commission of Economy and Informatization (https://jxt.zj.gov.cn/), including the time and location of policy implementation.
3. Control variables. The digital economy industry is essentially the result of the deep integration of traditional economy and the internet, and its development is influenced by the level of regional economic and social development, as well as the foundation and potential for regional informatization. Therefore, we collect the following control variables to mitigate the impact of these two factors on the digital economy industry development, including ln*Pgdp*, ln*Pop*, *Secondary*, *Tertiary*, *Fixed*, and *Patent*. ln*Pgdp* measured using per capita GDP 2005 constant RMB. Per capita GDP is an important indicator for measuring the level of regional economic and social development. As a product of the combination of economic activities and the internet, the digital economy industry development is inevitably influenced by the level of regional economic development [14, 27]. ln*Pop* measured using the year-end total population of a region. When the size of the population is in line with the scale of the digital economy, population expansion plays a positive role in the development of the digital economy industry. Population agglomeration reduces the shared costs of production factors and infrastructure, enhances resource matching, and accelerates knowledge and technology spillovers [28]. *Secondary* is measured by the ratio of the value-added of the secondary industry to the GDP; *Tertiary* is measured by the ratio of the value-added of the tertiary industry to the GDP. *Fixed* is measured by the ratio of the fixed assets investment to the GDP. With the restructuring and optimization of industrial structure, there is an increasing demand for traditional industries to embark on digital transformation. This shift offers greater opportunities for the development of the digital economy industry [29]. Capital is a pivotal factor in production, playing a crucial role in the form of both direct and indirect investments, ultimately contributing to digital economic products, labor, intermediate goods in the production process, and financial assets [14, 30]. The centralization of capital facilitates enterprises in utilizing large-scale production machinery, thereby generating economies of scale. *Patent* is measured using number of authorized patents. Regional innovation is a key influencing factor in the development of the digital economy industry. The stronger the regional innovation capability, the faster the development of the digital economy industry [31]. The data on control variables come from the Zhejiang Provincial Statistical Yearbook, statistical yearbooks of prefecture-level cities in Zhejiang, and the statistics communique on the national economic and social development of prefecture-level cities in Zhejiang.

We use panel data for 89 county-level administrative regions in Zhejiang from 2005 to 2020. The county-level administrative region is an administrative region with the same administrative status as the county. This paper includes three categories: municipal district, county-level city, and county. In addition, during the period from 2005 to 2020, some regions of Zhejiang underwent administrative boundary adjustments, which altered the names and codes of the administrative divisions. However, this did not affect the statistical data. In the data organization for this paper, the names and codes of the county-level divisions are based on the codes as of the end of 2020, to facilitate the merging of data for analysis. Table 3 shows the descriptive statistics of variables.

**Table 3:** Descriptive statistics of variables.

Variables	Obs.	Mean	Std. Dev.	Min.	Max.
*Y*	1424	-0.024	0.955	-0.434	5.489
*IEDD*	1424	0.079	0.269	0.000	1.000
*SISI*	1424	0.093	0.290	0.000	1.000
ln*Pgdp*	1424	0.989	0.565	-0.471	2.434
ln*Pop*	1424	3.884	0.591	2.084	5.024
*Secondary*	1424	0.477	0.124	0.093	0.747
*Tertiary*	1424	0.456	0.131	0.238	0.911
*Fixed*	1424	0.561	0.253	0.136	1.656
*Patent*	1424	31.296	87.763	0.060	743.44

## Spatial-temporal changes of digital economy industry

### Spatial pattern of digital economy industry

**Fig 1:**
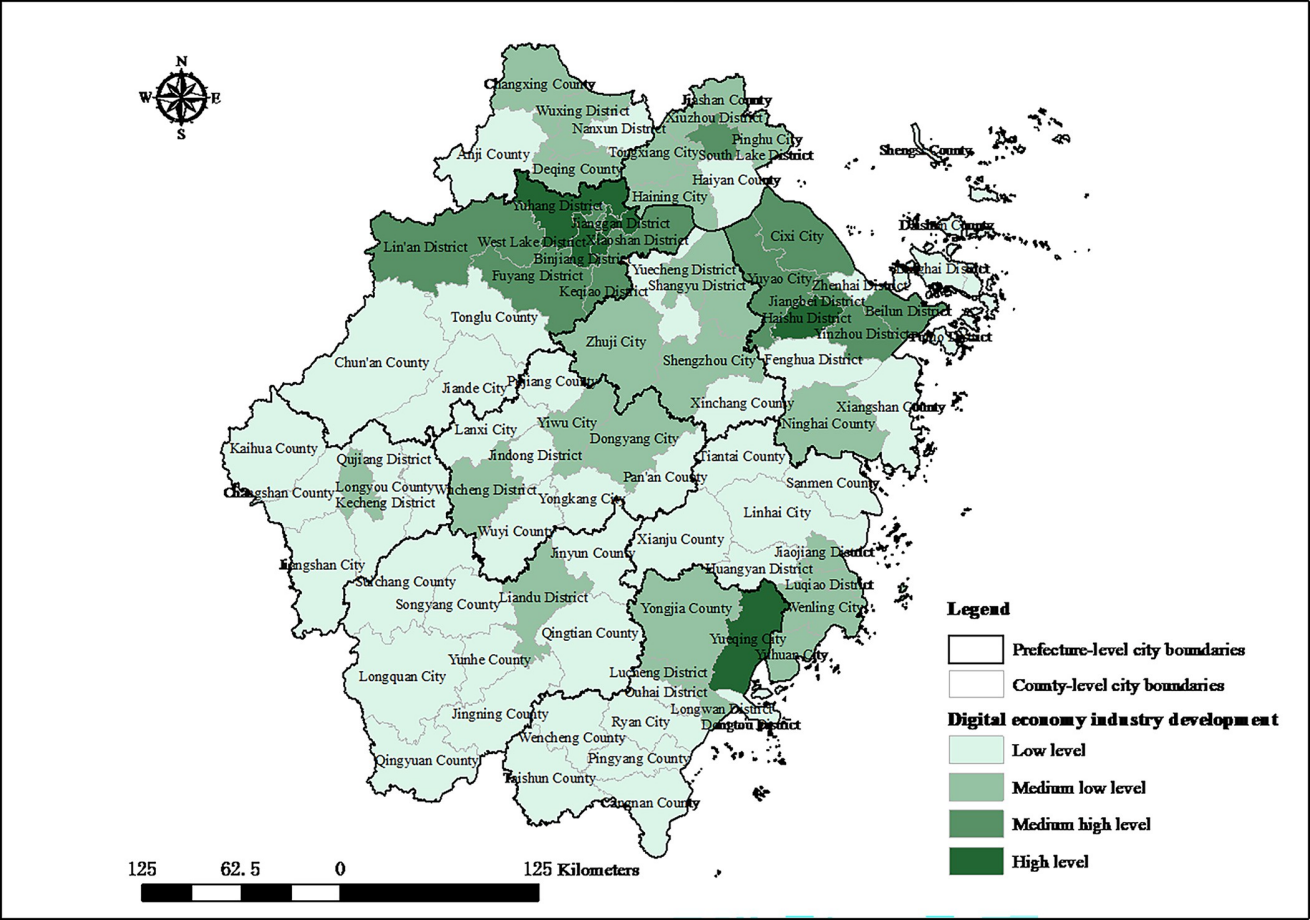
Spatial distribution of digital economy industry development index in Zhejiang county-level administrative regions in 2005. Source: created by the author based on the base map of Zhejiang which comes from the China National Catalogue Service For Geographic Information (https://www.webmap.cn/).

**Fig 2:**
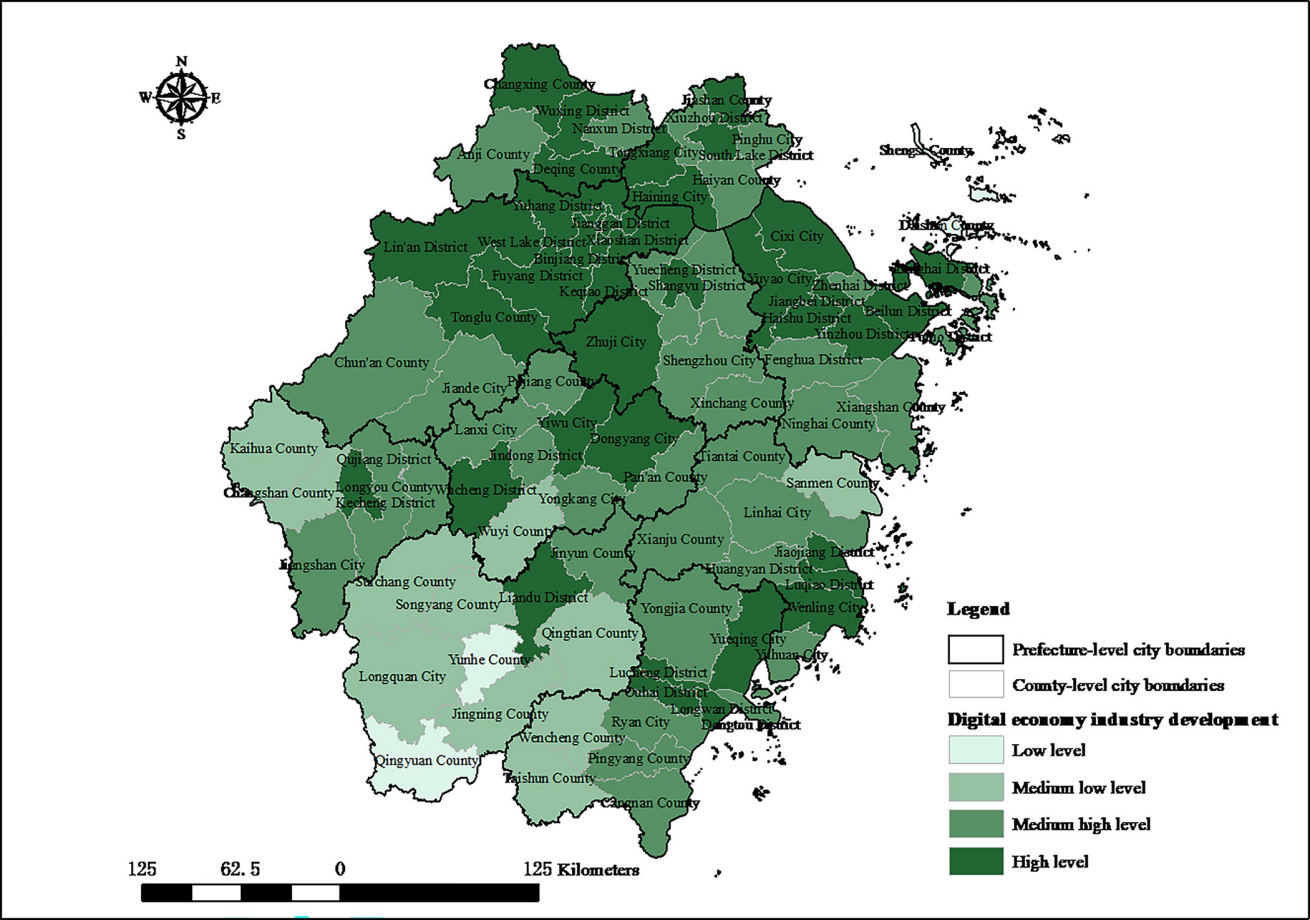
Spatial distribution of digital economy industry development index in Zhejiang county-level administrative regions in 2020. Source: created by the author based on the base map of Zhejiang which comes from China National Catalogue Service For Geographic Information (https://www.webmap.cn/).

The categorization of the digital economy industry development index is established using quartile groupings. Specifically, the index is segmented into four distinct stages: low, medium low, medium high, and high. These stages correspond to the 25th, 50th, and 75th percentiles, respectively, providing a structured framework to compare the development level of the digital economy industry across different years or regions. The spatial distribution of the digital economy industry development level in Zhejiang in 2005 and 2020 is shown in Figs 1 and 2, respectively. The development of digital economy industry in Zhejiang is on an upward trajectory, characterized by a pattern of clustered distribution that exhibits marked regional disparities. In 2005, Hangzhou, Wenzhou, and Ningbo emerged as the leading cities in Zhejiang province in terms of digital economy industry development, showcasing the highest levels of sectoral agglomeration. In 2005, the registered capital of digital economy enterprises in Hangzhou, Wenzhou, and Ningbo reached 131.894 billion RMB, 36.492 billion RMB, and 35.604 billion RMB, respectively. These figures accounted for 50.95%, 14.10%, and 13.76% of the Zhejiang province’s total registered capital, respectively. This data clearly indicates that Hangzhou is at forefront of digital economy industry development within Zhejiang. In 2020, Zhejiang saw a significant improvement in the development level of its digital economy industry. It is evident that the implementation of IEDD and SISI policies corresponds with a swifter improvement in the digital economy industry’s development compared to regions without such policies. Notably, the digital economy’s development in regions such as West Lake District of Hangzhou City, Yueqing City of Wenzhou City, and Yinzhou District of Ningbo City has seen rapid enhancement. The digital economy industry’s development hub, centered around Hangzhou, Wenzhou, and Ningbo, has been expanding. Additionally, the agglomeration of digital economy industry has also started to emerge in Jiaxing and Jinhua. Currently, the digital economy industrial clusters among county-level administrative regions have been establishing a pattern of coordinated spatial development, with Hangzhou Bay as the center hub, and Wenzhou, Ningbo, Jiaxing, and Jinhua emerging as secondary centers. In summary, there are significant variances in the developmental trajectories of the digital economy across county-level administrative regions in Zhejiang. The extent to which these disparities are attributable to the implementation of digital economy policies requires additional investigation.

### Spatial correlation analysis of digital economy industry

**Fig 3:**
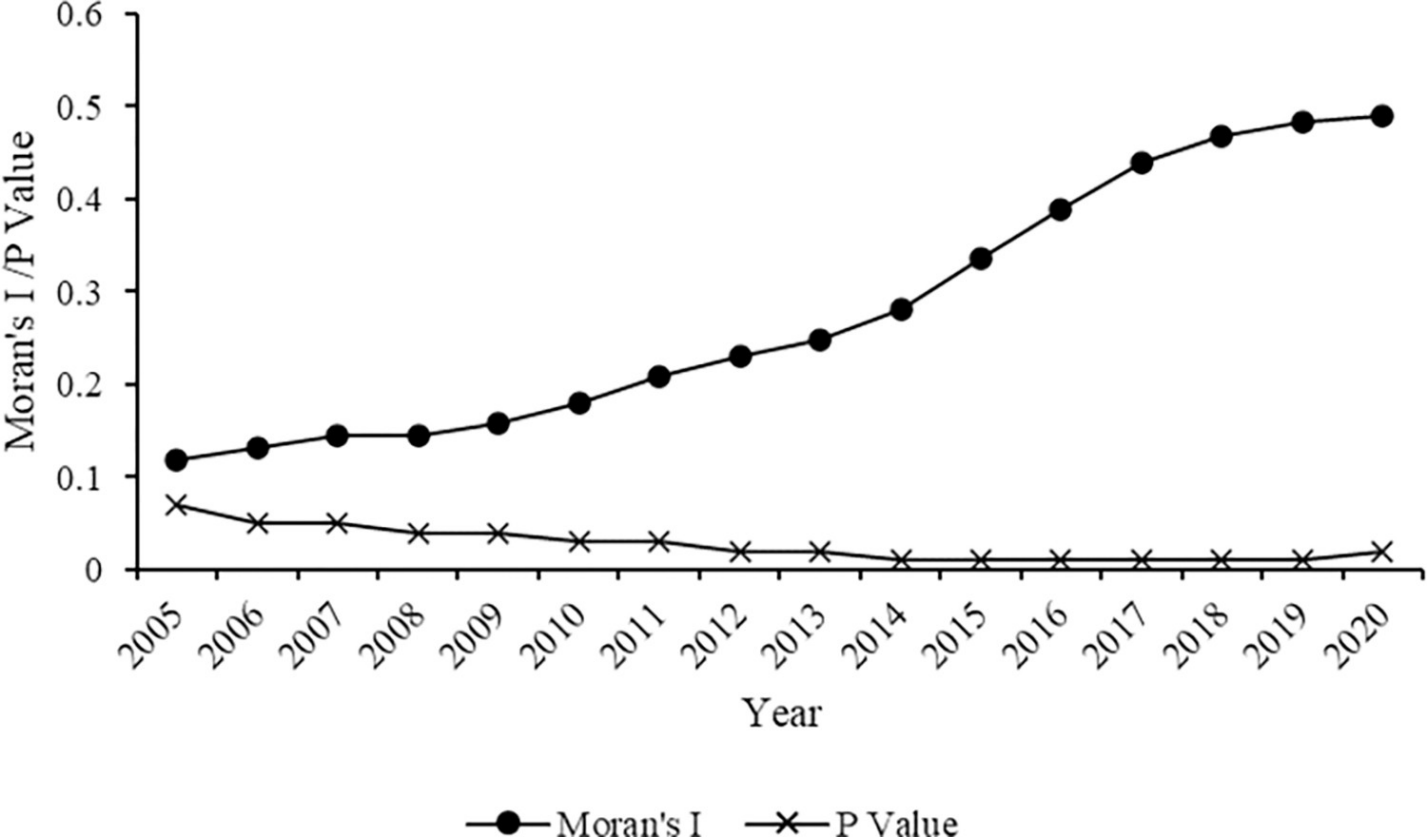
Moran’ s I values of digital economy industry development in Zhejiang county-level administrative regions from 2005 to 2020.

We further utilize the global spatial autocorrelation coefficient to quantify the extent of spatial clustering of the digital economy industry development in Zhejiang. Fig 3 illustrates Moran’s I values of digital economic industry development in Zhejiang from 2005 to 2020. Moran’s I values are consistently positive, exhibiting a rising trajectory from 2005 to 2020. This indicates that the development of digital economy industry in Zhejiang county-level administrative regions possesses marked positive spatial correlation traits, with the spatial agglomeration effect intensifying over time.

**Fig 4:**
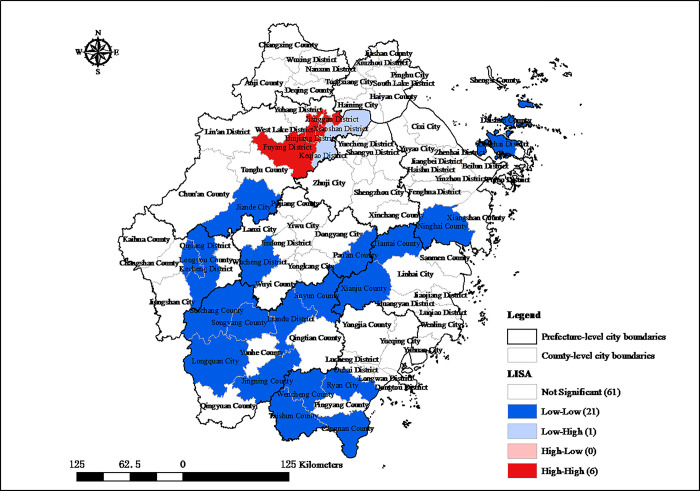
Local autocorrelation of digital economy industry development in Zhejiang county-level administrative regions in 2005. Source: created by the author based on the base map of Zhejiang which comes from China National Catalogue Service For Geographic Information (https://www.webmap.cn/).

**Fig 5:**
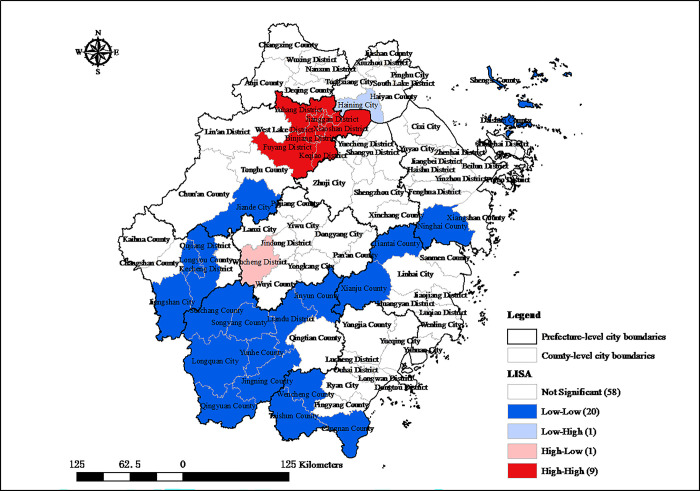
Local autocorrelation of digital economy industry development in Zhejiang county-level administrative regions in 2020. Source: created by the author based on the base map of Zhejiang which comes from China National Catalogue Service For Geographic Information (https://www.webmap.cn/).

To further scrutinize the local spatial autocorrelation of the digital economy industry development in Zhejiang, Local Indicators of Spatial Association (LISA) cluster maps are created of the development of the digital economy industry in 2005 and 2020, as depicted in Figs 4 and 5, respectively. In 2005, the central urban area of Hangzhou was characterized by a significant “high-high” local spatial autocorrelation, indicating a concentration of high-value digital economy activity. Conversely, the southwestern part of Zhejiang demonstrated a “low- low” local spatial autocorrelation, reflecting a cluster of areas with lower industry values. Over 15 years, the “high-high” clusters within Hangzhou have expanded from 6 to 9 county-level administrative regions, indicating growth in these high-value industry zones. Despite this expansion, a “low-low” clusters have persisted, with their central gravity shifting westward. These patterns reveal that while Hangzhou are significantly ahead, there are pronounced structural disparities in the spatial distribution of digital economy industry across Zhejiang.

## Empirical results

### Baseline regression

In this section, we study the impact of digital economy policies on the digital economy industry development. Table 4 reports the baseline regression results from Eq (1). We begin with a simplest specification by only controlling for IEDD and SISI policies, county-level administrative region fixed, and year fixed effects. The result is shown in column (1) of Table 4. The coefficients of *IEDD* and *SISI* are significantly positive at 1% level which suggest that and the two policies both have positive effects on the digital economy industry development. Moreover, the effect of the SISI policy is significantly higher than that of IEDD.

**Table 4:** The baseline estimation results.

	(1)	(2)	(3)
*IDEE*	0.405*** (8.39)	0.220*** (4.79)	0.259*** (6.24)
*SISI*	0.607*** (13.27)	0.413*** (9.47)	0.414*** (10.16)
ln*Pgdp*		0.193*** (2.83)	-0.019 (-0.29)
ln*Pop*		0.686*** (8.31)	0.313 (3.83)
*Secondary*		-0.713*** (-3.95)	-0.552*** (-3.25)
*Tertiary*		1.369*** (6.51)	0.627*** (3.07)
*Fixed*		-0.063 (-1.11)	-0.052 (-0.83)
*Patent*		0.006*** (6.57)	0.005*** (5.28)
Constant	-0.263*** (-7.23)	-0.318*** (-7.33)	-2.578*** (-6.07)
County fixed	Yes	Yes	Yes
Year fixed	Yes	Yes	Yes
City fixed × Year fixed	No	No	Yes
*R* ^2^	0.542	0.554	0.702
Obs.	1424	1424	1424

*Notes*: *t*-values in parentheses; ***p<0.01, **p<0.05, and *p<0.1.

In column (2) of Table 4, we add a series of control variables, the coefficients of *IEDD* and *SISI* remain significantly positive at 1% level and the coefficient values of *IEDD* and *SISI* are significantly lower than those of column (1), which implies that these control variables significantly contribute to explaining the digital economy industry development. There are differences in these control variables between county-level administrative regions with demonstration base and those without demonstration base. Without controlling control variables could lead to an overestimation of the policy effects.

In column (3) of Table 4, we further add the interaction term of city fixed effect and year fixed effect. The coefficients of *IEDD* and *SISI* remain significantly positive at 1% level and the estimated coefficients of *IEDD* and *SISI* have slightly decreased compared with column (2). This indicates that county-level administrative regions in the same city have similar changing trends in digital economy industry development, and there are differences in the regional distribution between county-level administrative regions with and without demonstration base. Failure to control this difference in column (2) will overestimate the implementation effect of the IEDD and SISI policies. To sum up, in column (3), multiple factors such as the region fixed effect, the year fixed effect, and the interaction term between them are all controlled at the same time, so the design of column (3) is more rigorous. Although the estimated coefficients of the two policies are relatively small in column (3), the results still show that the two policies have significantly positive impacts on the development of the digital economy industry. Then, we conduct an analysis based on the estimated results in column (3). According to the results of column (3) in Table 4, the IEDD and SISI policies implementation increase the county-level administrative region’s digital economy industry development level by an average of 25.9% and 41.1%, respectively. This verifies Hypothesis 1.

Parallel trend test

The parallel trend assumption is the premise of using DID model to test the impact of the IEDD and SISI policies on the digital economy industry development in Zhejiang. We draw on the research of Chava et al. (2013) [32] to conduct the following model for the parallel trend test:

$$\begin{array}{l} Y_{ijt}=\theta_{0}+\alpha_{-b}\sum_{b=-4}^{-2}IDEE_{-b,ijt}+\alpha_{a}\sum_{a=0}^{4}IDEE_{a,ijt}+\beta_{-b}\sum_{b=-4}^{-2}SISI_{-b,ijt}\\ \;+\beta_{a}\sum_{a=0}^{4}SISI_{a,ijt}+\lambda X_{ijt}+\gamma_{j}+\mu_{t}+\varepsilon_{it} \end{array}$$ (2)

where *b* represents the year before the implementation of IEDD and SISI policies; *a* represents the year after the implementation of IEDD and SISI policies, *a* = 0 indicates the year in which IEDD and SISI policies are implemented. In Eq (2), the first year before the implementation of IEDD and SISI policies is taken as the reference year, so the variables *IEDD*_-1_ and *SISI*_-1_ are omitted in the model. The coefficients *α* and *β* measure the change in digital economy industry development in any year relative to the implementation reference year of IEDD and SISI policies.

**Fig 6:**
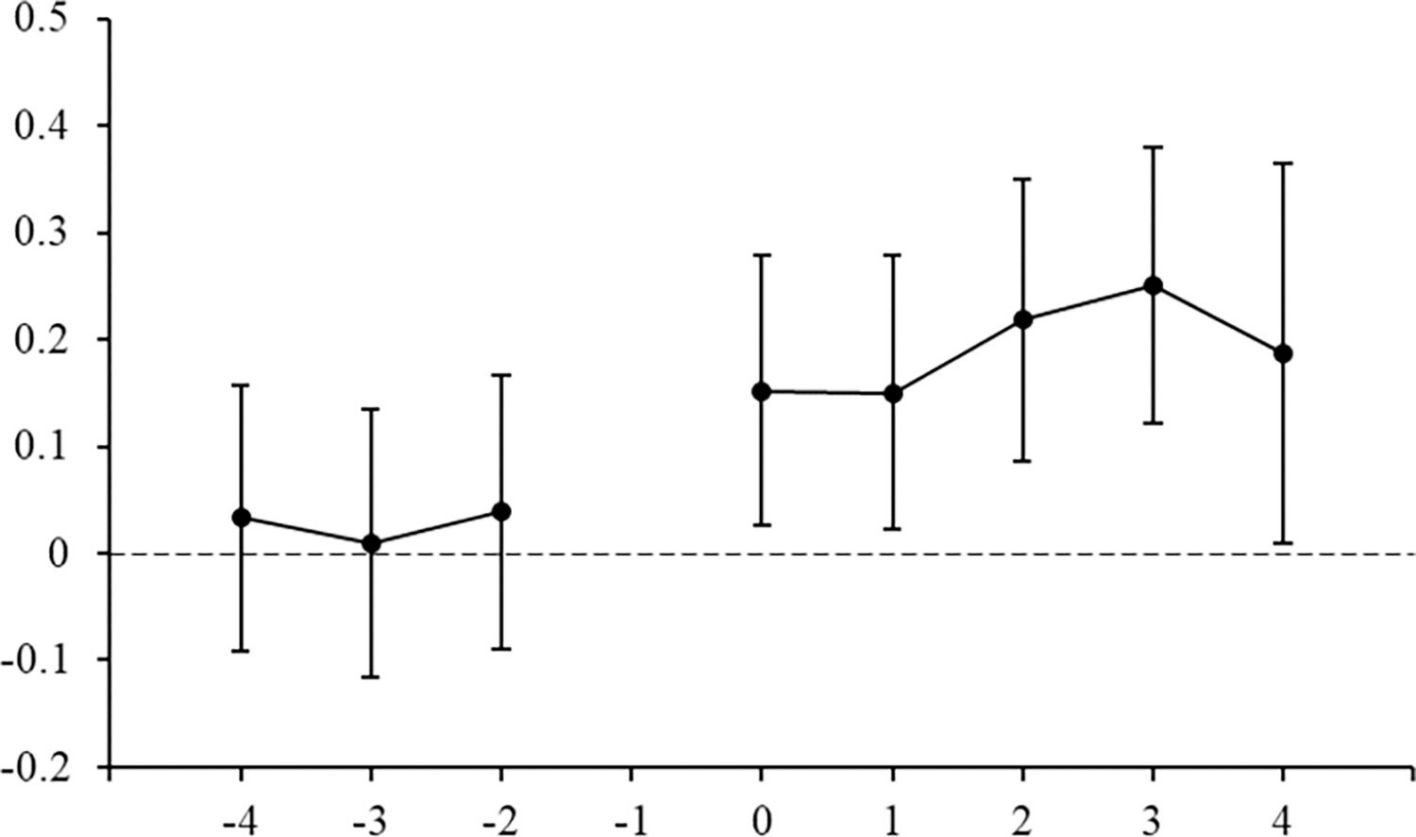
Parallel trend test for IEDD policy.

**Fig 7:**
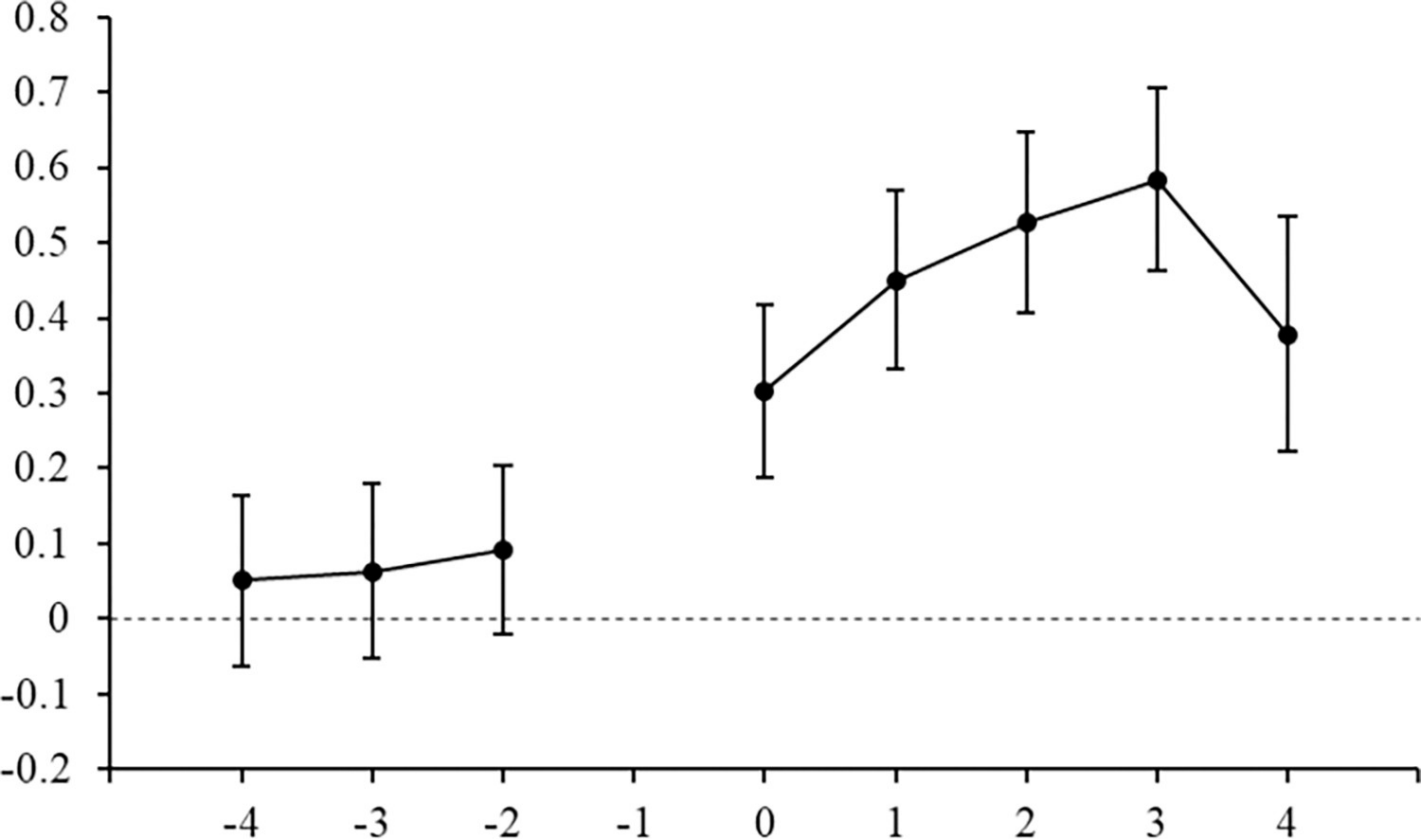
Parallel trend test for SISI policy.

Figs 6 and 7 illustrate the estimation results of coefficients *α* and *β* for IEDD or SISI policies, respectively. In Figs 6 and 7, the horizontal axis represents the years before and after the implementation of the IEDD or SISI policies, and the vertical axis represents the estimated coefficient values and confidence intervals. In Fig 6, in the 4th, 3rd, and 2nd years before the establishment of IEDD policy, the regression coefficients of *IEDD* are near 0 and not significant. This suggests that there is no significant difference between the treatment group and control group before the implementation of IEDD policy, that is, it meets the parallel trend assumption. In the 1th year, 2nd year, 3rd year, and 4th year after the establishment of IEDD policy, the development level of digital economy industry in the treatment group is significantly higher than that in the control group. Fig 7 shows similar results, that is, there is no significant difference between the treatment group and control group before the implementation of SISI policy, which also meets the parallel trend test.

### Robustness checks

#### Alternative dependent variables

In this section, we use alternative dependent variables for robustness checks. The level of digital economy industry development is measured using four secondary indicators: the number of digital economy enterprises (*Enterprises*), total registered capital (*Capital*), total outward investment (*Investment_O*), and total accepted investment (*Investment_A*), respectively. The estimated results in Table 5 show that the coefficients of *IEDD* and *SISI* are significantly positive at 1% level, except for the coefficient of *IEDD* in column (4) which is not significant. These results reaffirm our previous findings. The implementation of IEDD and ISIS policies promotes the expansion of digital economy enterprise scale and investment.

**Table 5:** Alternative dependent variables.

	(1) *Enterprises*	(2) *Capital*	(3) *Investment_O*	(4) *Investment_A*
*IDEE*	0.548*** (10.32)	0.514*** (9.86)	0.118*** (4.61)	0.025 (1.23)
*SISI*	0.572*** (10.93)	0.571*** (11.14)	0.092*** (3.62)	0.162*** (8.05)
ln*Pgdp*	0.379*** (4.47)	0.189** (2.28)	0.149*** (3.63)	0.112*** (3.44)
ln*Pop*	1.040*** (9.93)	0.630*** (6.14)	0.472*** (9.33)	0.198*** (4.91)
*Secondary*	-0.572*** (-2.63)	-0.398* (-1.86)	0.398*** (3.78)	-0.243*** (-2.90)
*Tertiary*	1.433*** (5.47)	1.24*** (4.86)	0.040 (0.31)	0.596*** (5.90)
*Fixed*	-0.077 (-0.97)	-0.119 (-1.54)	-0.099** (-2.58)	0.028 (0.92)
*Patent*	0.011*** (9.58)	0.011*** (9.90)	0.002*** (4.67)	0.002*** (5.28)
Constant	-6.539*** (-12.01)	-4.685*** (-8.78)	-2.912*** (-11.07)	-1.658*** (-7.89)
County fixed	Yes	Yes	Yes	Yes
Year fixed	Yes	Yes	Yes	Yes
City fixed × Year fixed	Yes	Yes	Yes	Yes
*R* ^2^	0.810	0.770	0.539	0.601
Obs.	1424	1424	1424	1424

*Notes*: *t*-values in parentheses; ***p<0.01, **p<0.05, and *p<0.1.

#### Eliminate the influence of leading enterprises

Considering that Alibaba’s headquarters is in Hangzhou, it may have demonstration and agglomeration effects on the digital economy industry development in Hangzhou. To eliminate the potential impact of Alibaba, we exclude Shangcheng, Xiacheng, Xihu, Jianggan, Gongshu, Binjiang, Yuhang, Xiaoshan, Fuyang, and Lin’an Districts from the sample. The ten districts are all under the jurisdiction of Hangzhou. Column (1) in Table 6 reports the estimation results. Compared with the results of column (4) in Table 4, the coefficient values of *IEDD* and *SISI* are obviously lower, indicating that as the foremost enterprise in digital economy, Alibaba plays a significant role in propelling the digital economy industry in Zhejiang. If the influence of Alibaba is not excluded, the effects of IEDD and SISI policies will be overestimated. Nevertheless, the coefficients of *IEDD* and *SISI* are significantly positive at 1% level, which means that the IEDD and SISI policies significantly promote the digital economy industry development in Zhejiang, suggesting that the baseline regression results are robust.

**Table 6:** Robustness checks.

	(1)	(2)	(3)	(4)
*L*.*y*				0.803*** (4.16)
*IDEE*	0.254*** (10.99)	0.219*** (4.69)	0.218*** (5.69)	0.114* (1.95)
*SISI*	0.185*** (8.01)	0.378*** (8.37)	0.372*** (9.56)	0.184** (2.45)
ln*Pgdp*	0.140*** (3.27)	-0.130 (-1.35)	-0.131* (-1.88)	-0.102 (-1.10)
ln*Pop*	0.311*** (5.93)	0.190* (1.75)	0.289*** (3.57)	0.011 (0.07)
*Secondary*	-0.305*** (-3.19)	-0.606*** (-2.26)	-1.009*** (-5.51)	-0.217 (-1.56)
*Tertiary*	0.723*** (5.99)	0.786** (2.57)	0.676*** (3.43)	0.030 (0.13)
*Fixed*	-0.078*** (-2.29)	-0.296*** (-3.44)	-0.180*** (-2.73)	-0.058 (-1.13)
*Patent*	0.003*** (5.78)	0.002** (2.11)	0.001 (1.52)	0.001 (0.56)
Constant	-1.903*** (-6.33)	-1.858 (-3.04)	-1.954*** (-4.53)	0.192 (0.22)
County fixed	Yes	Yes	Yes	Yes
Year fixed	Yes	Yes	Yes	Yes
City fixed × Year fixed	Yes	Yes	Yes	Yes
*R* ^2^	0.707	0.683	0.703	
Obs.	1264	979	1296	1335
Hansen				1.000
AR (2)				0.101

*Notes*: *t*-values in parentheses; ***p<0.01, **p<0.05, and *p<0.1; Hansen statistics and AR (2) statistics are both *P* values.

#### Shorten the time span of the sample

IEDD and SISI policies have been implemented since 2015 and 2016, respectively. The time span of the baseline regression sample is 2005 to 2020, and the period before the implementation of policies is substantial. Therefore, we use the sample from 2010 to 2020 to repeat the estimation of Eq (1), and the results are shown in column (2) in Table 6. The coefficients of *IEDD* and *SISI* remain statistically significant and positive, suggesting that our findings remain robust.

#### Propensity Score Matching (PSM) regression

We further use the PSM method to conduct the robustness check. For the treatment group, we select a control group whose characteristics are as similar as possible to the treatment group to eliminate the sample selection bias. Then we repeat the estimation of Eq (1) using matched treatment and control groups, and the results are shown in column (3) in Table 6. The coefficients of *IEDD* and *SISI* remain significantly positive at 1% level.

#### Generalized Method of Moments (GMM) regression

Most of the county-level administrative regions that implement IEDD and SISI policies are those with rapid development of digital economy in recent years. Whether a region is selected as a demonstration base may be related to the local economic development, which may lead to the endogeneity issue of policy variables. We introduce the lagged term of the dependent variable into the model and conduct a GMM method to solve this endogeneity issue. The estimated results of GMM are shown in column (4) of Table 6. The coefficient of the lag item of the dependent variable is significantly positive, and the coefficients of the *IEDD* and *SISI* are also significantly positive, suggesting that the two policies have significant positive impacts on the digital economy industry development in Zhejiang, which reinforces our previous findings.

#### Placebo test

We draw on the research of Wang et al. (2021) [33] to randomly set the implementation year and the county-level administrative regions of demonstration bases for the placebo test. The model is set as follows:

$$\hat{\beta }=\beta +\gamma *cov(Policy\_it,\varepsilon \_it|X)/var(Policy\_it|X)$$ (3)

where *Policy* represents policy variables (*IEDD* or *SISI*); *X* represents a series of control variables; *β* denotes the unbiased estimator of the demonstration base; *γ* is correlation coefficient, if $\hat{\beta }$ equal to 0, then *γ* = 0, which means that the year and location of the randomly generated demonstration base have no impact on the digital economy industry development in Zhejiang. It further shows that in Eq (1), the effect of the two policies on promoting the rapid development of the digital economy industry in Zhejiang is actual.

**Fig 8:**
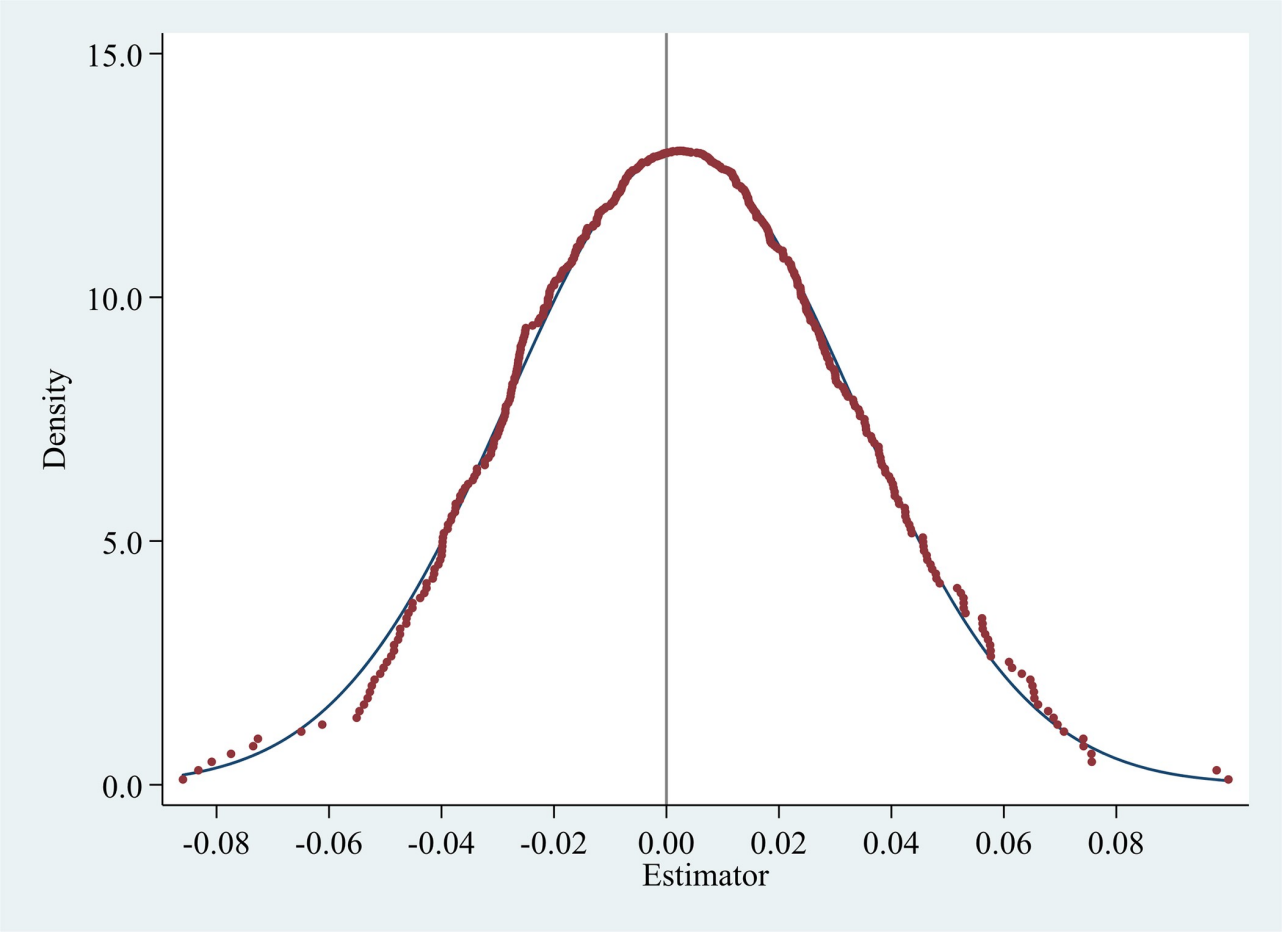
Placebo test for IEDD (the mean is 0.0025549).

**Fig 9:**
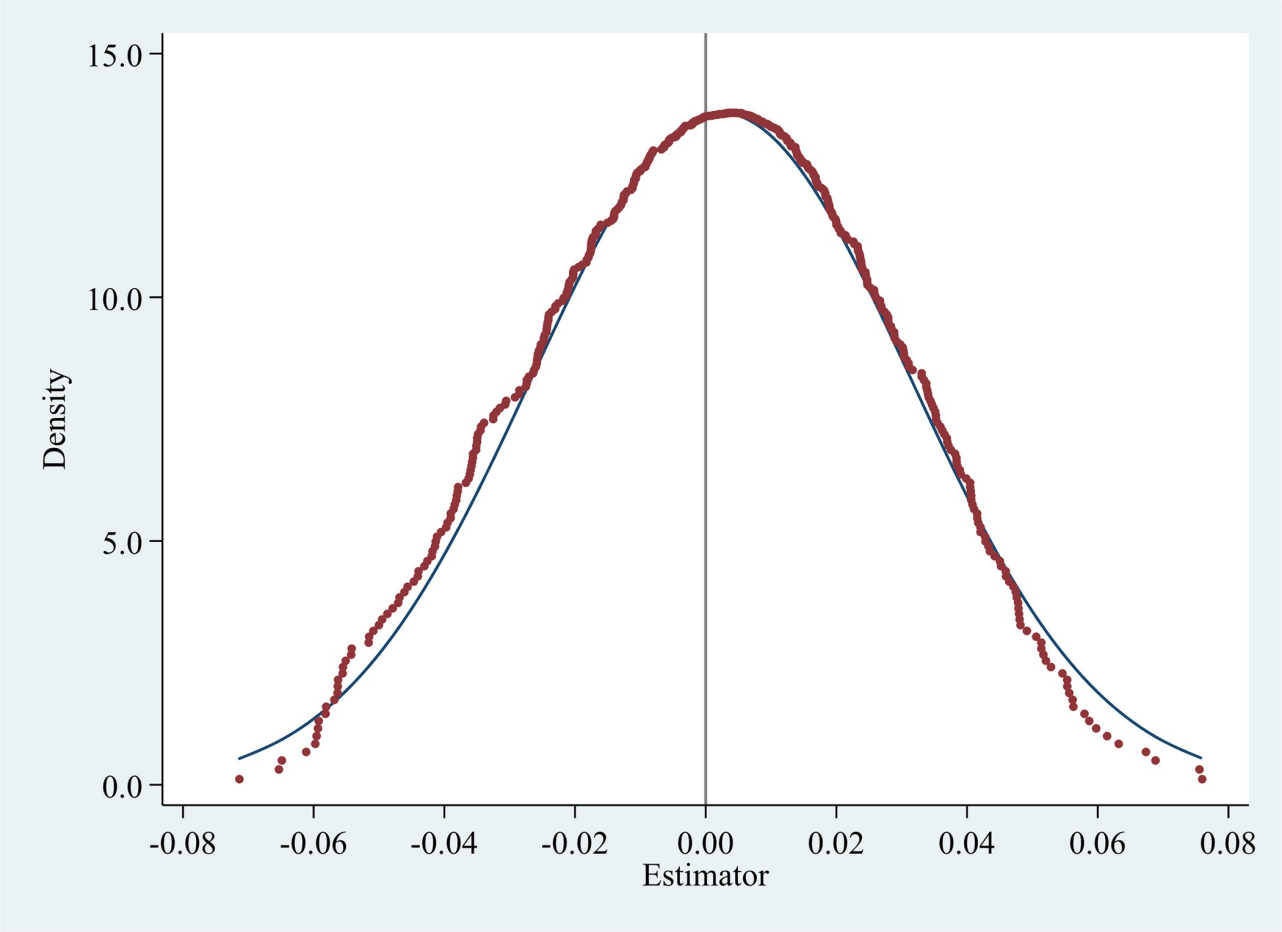
Placebo test for SISI (the mean is 0.0023603).

There are two batches of IEDD policy. The first batch set up 12 bases in 2015, and the second batch set up 10 bases in 2017. To make the placebo experiment consistent with the actual policy implementation, we randomly generate two policy implementation times, and randomly assign the same number of treatment group and control group at each policy time point. By doing so, we can construct a “fake” *IEDD* variable based on the random assignments of year and location in each county-level administrative region for policy implementation. We then conduct regressions in Eq (1) using this “fake” *IEDD* to replace the actual *IEDD* variable. To obtain consistent results, we repeat this random exercise 500 times. We plot the density of the estimated coefficients on “fake” *IEDD* in Fig 8. The placebo experiment of the same method is also carried out on the *SISI*. We plot the density of the estimated coefficients on “fake” *SISI* in Fig 9. If the regression coefficients of *IEDD* or *SISI* in the placebo test do not significantly deviate from zero, it suggests that the baseline regression results are robust. In Figs 8 and 9, the distribution of the estimated coefficients of the randomly generated treatment group is close to the normal distribution with the symmetry axis close to “0”. And the mean values are 0.003 and 0.002, respectively, which are significantly different from the estimated coefficients of the baseline regression (the estimated coefficients of *IEDD* and *SISI* in column (3) of Table 4 are 0.259 and 0.414, respectively). The results of this placebo test reaffirm that our findings are unlikely to be spurious.

#### Mechanism analysis

As noted above, it is also important to consider whether IEDD and SISI policies can promote the digital economy industry development by enhancing digital infrastructure and financial development. We examine whether they stimulate such developments, using the digital infrastructure (*Inter*) and financial development level (*Fina*) as intermediate mechanism variables. *Inter* is measured as Internet coverage, and *Fina* is measured as year-end loan balance of financial institutions. The results are shown in Table 7.

**Table 7:** Mechanism analysis.

	(1) *Y*	(2) *Inter*	(3) *Fina*	(4) *Y*
*IDEE*	0.415*** (11.40)	0.615*** (8.44)	0.282*** (4.75)	0.311*** (8.78)
*SISI*	0.310*** (8.35)	0.086 (1.16)	0.196*** (3.24)	0.274*** (7.85)
*Inter*				0.106*** (6.19)
*Fina*				0.139*** (6.61)
ln*Pgdp*	-0.164** (-2.58)	-0.051 (-0.40)	0.013 (0.13)	-0.160*** (-2.71)
ln*Pop*	-0.028 (-0.34)	0.414** (2.48)	0.766*** (5.65)	-0.179** (-2.26)
*Secondary*	-0.298* (-1.88)	-0.538* (-1.69)	-0.301 (-1.16)	-0.199 (-1.34)
*Tertiary*	0.244 (1.28)	0.190 (0.50)	1.187*** (3.81)	0.059 (0.33)
*Fixed*	-0.020 (-0.39)	0.1489 (1.45)	-0.198** (-2.37)	-0.008 (-0.17)
*Patent*	0.001*** (4.39)	0.002*** (5.08)	0.001*** (2.80)	0.001*** (2.83)
Constant	-0.957** (-2.23)	-2.484*** (-2.89)	-4.308*** (-6.16)	-0.093 (-0.23)
County fixed	Yes	Yes	Yes	Yes
Year fixed	Yes	Yes	Yes	Yes
City fixed × Year fixed	Yes	Yes	Yes	Yes
*R* ^2^	0.657	0.774	0.767	0.703
Obs.	1104	1104	1104	1104

*Notes*: *t*-values in parentheses

***p<0.01

**p<0.05, and

*p<0.1.

The coefficients of *IDEE* in Table 7, columns (2) and (3) are both significantly positive. This means that IDEE policy can improve the digital infrastructure and financial development. The coefficient of *SISI* in Table 7, column (2) is not significantly, but is significantly positive in Table 7, column (3). This indicates that SISI policy promotes financial development, but does not promote digital infrastructure. This is likely due to the significant investment and resource requirements associated with the construction of digital infrastructure. Establishing software and information service industry bases can offer specific support and guidance, but it may not fully address all the funding and resource requirements at present.

Then, we additionally introduce *Inter*, and *Fina* variables in Eq (1), and the estimated coefficients of *IDEE*, *SISI*, *Inter*, and *Fina* are all statistically significant at 1% level and positive. In addition, the coefficient values of *IDEE* and *SISI* in column (4) are smaller than those in column (1). (i.e., 0.311 and 0.274 after introducing *Inter*, and *Fina* variables in column (4), respectively, compared to 0.415 and 0.310 for result in column (1)). This shows that IEDD policy does promote the development of the digital economy industry by promoting digital infrastructure and financial development; SISI policy can promote the development of the digital economy industry by promoting financial development.

The digital infrastructure and financial development provide technological and capital support for the digital economy industry, collectively driving the development of the digital economy industry. A robust digital infrastructure can enhance the efficiency of data transmission and processing, reducing the costs for businesses and individuals to access and utilize digital resources. This, in turn, creates a more favorable environment for the development of the digital economy industry. Financial institutions can provide funding support to the digital economy industry through various financial instruments such as loans, venture capital, and securities financing. This assistance aids companies in research and development investment, expanding production scale, and market expansion, thus driving the rapid development of the digital economy industry.

### Heterogeneity analysis

#### Quantile regression

To describe the distribution characteristics of the impact of IEDD and SISI policies on the digital economy industry development more comprehensively, we conduct the quantile regression model. This approach is superior compared to a traditional regression on outlier handling, as it can estimate the conditional median and other conditional quantiles of the dependent variable; in contrast, the traditional regression can estimate the conditional average of the dependent variable only. The panel quantile regression model can be expressed as follows:

$$Q_{Y_{it}}^{\left(\tau \right)}(\tau |Z_{it})=\alpha_{i}+Z_{it}^{T}\beta (\tau )+\varepsilon_{it},i=1,2,\ldots ,N,t=1,2,\ldots ,T$$ (4)

where *α*_*i*_ represents fixed effect, which does not change with the variation in quantile; *Y*_*it*_ and *Z*_*it*_ represent the dependent and independent variables, respectively; *Z*_*it*_ includes *IEDD*, *SISI*, and a series of control variables; *τ* denotes the quantile; *ε*_it_ is the random error term. In particular, we estimate Eq (4) at the 25%, 50%, and 75% quantile.

Table 8 reports the panel quantile regression results, and the results show the evidence of heterogonous effects across county-level administrative regions with different development levels of digital economy industry. The coefficients of the *IEDD* and *SISI* are both significantly positive, and gradually increase with the increase of the quantile. That suggests that the implementation of IEDD and SISI policies in regions with good foundations for the digital economy has more obvious promotion effects on the digital economy industry development. The IEDD and SISI policies greatly stimulate the development potential of county-level administrative regions with a certain industrial foundation. The IEDD policy focuses on the digital economy development as a whole in the region, cultivates leading industries, and improves technological content. The SISI policy pays attention to the development of software and information services industry, which requires superior internet infrastructure, so it works better in county-level administrative regions with a good foundation for the digital economy industry development.

**Table 8:** Quantile regression results.

	(1)	(2)	(3)
	q25	q50	q75
*IEDD*	0.137*** (10.73)	0.213*** (5.94)	0.271*** (10.56)
*SISI*	0.097*** (7.74)	0.181*** (5.15)	0.258*** (10.21)
ln*Pgdp*	0.006 (0.29)	0.068 (1.19)	0.098** (2.41)
ln*Pop*	0.157*** (6.26)	0.324*** (4.59)	0.336*** (6.64)
*Secondary*	-0.037 (-0.71)	-0.069 (-0.47)	-0.135 (-1.28)
*Tertiary*	-0.108* (1.72)	0.235 (1.33)	0.317** (2.51)
*Fixed*	-0.021 (-1.11)	0.006 (0.12)	-0.006 (-0.15)
*Patent*	0.001*** (4.23)	0.002** (2.28)	0.001*** (2.72)
Constant	-1.049*** (-7.41)	-1.597*** (-4.02)	-1.632*** (-5.73)
County fixed	Yes	Yes	Yes
Year fixed	Yes	Yes	Yes
City fixed × Year fixed	Yes	Yes	Yes
*R* ^2^	0.661	0.720	0.827
Obs.	1424	1424	1424

*Notes*: *t*-values in parentheses

***p<0.01

**p<0.05, and

*p<0.1.

#### Group regression

Considering that there are great differences in the economic development, resource endowment, and industrial structure between the municipal district and the surrounding counties (county-level cities), we conduct group regression for the municipal district sample and the county (county-level city) sample. The empirical results are shown in Table 9. The columns (1) and (2) in Table 9 are the regression results of the district sample and county (county-level city) sample, respectively. In column (1), the coefficient of *SISI* is significantly positive, while the coefficient of *IEDD* is positive but not significant. It indicates that the SISI policy significantly promotes the digital economy industry development in districts, while the IEDD policy does not. In column (2), the coefficients of *IEDD* and *SISI* are both significantly positive, with the coefficient value of *IEDD* greater than that of *SISI*. It indicates that the IEDD and SISI policies promote the digital economy industry development in counties (county-level cities), and IEDD policy plays a pivotal role.

**Table 9:** Group regression results.

	(1)	(2)
	District sample	County and county-level city sample
*IEDD*	0.099 (1.14)	0.381*** (11.81)
*SISI*	0.722*** (7.86)	0.089** (2.49)
ln*Pgdp*	-0.403*** (-2.99)	0.107* (1.78)
ln*Pop*	-0.293* (-1.78)	0.405** (4.57)
*Secondary*	-1.078*** (-2.96)	-0.219 (-1.60)
*Tertiary*	0.324 (0.80)	0.560*** (2.83)
*Fixed*	-0.190 (-1.39)	0.024 (0.50)
*Patent*	0.005** (2.46)	0.002*** (3.34)
Constant	0.731 (0.83)	-2.330*** (-5.14)
County fixed	Yes	Yes
Year fixed	Yes	Yes
City fixed × Year fixed	Yes	Yes
*R* ^2^	0.786	0.656
Obs.	592	832

*Notes*: *t*-values in parentheses

***p<0.01

**p<0.05, and

*p<0.1.

The reason is that the internet infrastructures in districts are more complete, which meets the conditions for software information service industry development. In addition, the digital economy industry in Zhejiang is dominated by the service industry. In 2021, the registered capital of the digital economy service industry in Zhejiang is 4.5 times that of the manufacturing industry, and this gap is more prominent across districts. Therefore, the effect of the SISI policy in district is remarkable. Compared with districts, the internet infrastructure in counties (county-level cities) is backward. Counties (county-level cities) need to rely on the IEDD policy to prioritize the leading industries’ development, continuously improve the digital economy infrastructure, and enhance the vitality of innovation and entrepreneurship.

## Concluding remarks

The development of the digital economy is transformative for the way Chinese enterprises operate, fostering high-quality development of the Chinese economy. Zhejiang has been designated as the first common prosperity demonstration zone established by the Chinese government. Utilizing the digital economy to drive high-quality economic development in Zhejiang and enhance resident income bears substantial theoretical and practical significance. First, we collect data on digital economy enterprises in Zhejiang and analyze the spatial-temporal characteristics of the digital economy industry development in Zhejiang from 2005 to 2020. Then, based on the implementation of the IEDD and SISI policies, we use the quasi-natural experiment framework to assess the impact of these policies on the digital economy industry development. We find that there are significant spatial disparities in the digital economy industry development across county-level administrative regions of Zhejiang. Hangzhou is the center of the digital economy, and the digital economy industry is highly agglomerated, while other regions have not yet shown obvious high-high agglomeration. The IEDD and SISI policies promote the digital economy industry development in Zhejiang, enhancing the average development level of the digital economy industry by 25.9% and 41.4%, respectively. This finding remains robust following a series of robustness checks. In addition, IEDD policy promote the digital economy industry development by enhancing digital infrastructure and financial development; SISI policy can promote the development of the digital economy industry by promoting financial development. The results of heterogeneity analysis show that the IEDD policy promotes the digital economy industries development in counties (county-level cities), and the SISI plays a significant role in promoting the digital economy industry development in districts. The promotion effect of IDEE and SISI policies on the digital economy industry development increases with the improvement of the regional digital economy foundation.

Our findings show that the IEDD and SISI policies have important effects in promoting the digital economy industry development in Zhejiang. Furthermore, our results suggest that to further improve the development of digital economy in Zhejiang, the local government needs to work hard to eliminate the digital divide. As an emerging economic form in Zhejiang, the digital economy industry has given priority to development in Hangzhou and has developed rapidly in recent years. However, the gap between regions has also gradually increased, and the spatial structure is imbalanced. The local government should actively guide and promote the multi-centered and networked development of the digital economy industry in Zhejiang, so as to achieve balanced development in all regions. In addition, the local government should pay full attention to the leading role of top digital economies companies such as Alibaba and NetEase to promote the formation of a healthy and orderly digital economy industry ecology.

Considering that increasing the accumulation of human capital in industries related to the digital economy is conducive to the digital economy development, the supporting policies of digital economy should focus on improving human capital while providing financial support. The local government should promote the accumulation of human capital in the digital economy industry, especially in the regions with a weaker foundation of digital economy industry, strengthen the flow and exchange of talents among regions, and give full play to the leading role of regions such as Hangzhou, Ningbo, and Wenzhou.

In order to promote the development of digital economy industry, the local government should further improve the construction of digital platform. The local governments should continue to play the role of policies to guide the digital economy industry development, and constantly transform the policy advantage into industrial advantage. In addition, the government should actively build a fair, open and transparent digital governance platform for digital economy enterprises. In order to promote fair competition among enterprises and protect the legitimate rights and interests of consumers and workers, the government should create a market-oriented, legal and international business environment for enterprises by increasing the provision of digital governance and digital ecology and other government public goods such as data rights, security, transaction, and sharing.

In summary, we find that policy advantages can be transformed into industrial advantages, and we fully discuss the heterogeneity impact of policies on the digital economy. A possible extension for this study could be to identify channels, which can better help us understand how policies work. In addition, while the digital economy is booming, there is also a digital economy gap, the problem of uneven regional development is prominent, and large-scale monopoly enterprises have also emerged. One important future direction of the digital economy is digital governance policies such as anti-monopoly and fair competition, so as to promote the free and orderly flow of data, optimize the fair competition system of the digital economy, and improve the level of social welfare.

This paper contributes to the literature by highlighting that policy advantages can be transformed into industrial advantages. However, several aspects still need to be reconsidered by future research. First, there is a need for enhanced data support. Due to data accessibility constraints, comprehensive measurement of the digital economy’s development in Zhejiang counties through relevant indicators is challenged. Furthermore, statistical limitations regarding relevant indicators across 89 counties in Zhejiang preclude the use of some control variables. If we have more data, we can test more mechanisms, such as human capital. Nevertheless, with the Zhejiang Provincial Bureau of Statistics initiating data collection on digital economy-related industries at the city and county levels, there is an anticipation of more precise outcomes in future research.

## Supporting information

S1 Data Appendix A. We provide supplementary data for this paper here if you need it.
(XLSX)
